# Application of tele-ophthalmology in remote diagnosis and management of adnexal and orbital diseases

**DOI:** 10.4103/0301-4738.55078

**Published:** 2009

**Authors:** Malay Verma, Rajiv Raman, Ravindra E Mohan

**Affiliations:** Medical & Vision Research Foundation, Sankara Nethralaya, 18, College Road, Chennai-600 006, India

**Keywords:** Adnexal diseases, orbit, tele-ophthalmology

## Abstract

**Purpose:**

To assess the feasibility of making a diagnosis of adnexal and orbital diseases by Tele-ophthalmological means.

**Materials and Methods:**

Tele-consultation for eye diseases was done for 3497 patients from remote areas of Tamilnadu as part of the rural tele-ophthalmology project of a tertiary eye care hospital during a period of nine months from October 2004 to June 2005. These patients were comprehensively examined on-site by optometrists. Using digitized images sent by store and forward technique and videoconferencing, the ophthalmologist made a diagnosis and advised treatment.

**Results:**

Adnexal or orbital diseases were detected in 101 out of 3497 patients (2.88%). Medical treatment was advised to 13 of 101 patients (12.8%). Surgery was advised in 62 of 101 patients (61.28%) whereas 18 of 101 patients (17.8%) required further investigations at a tertiary center.

**Conclusion:**

It was feasible to apply the satellite based tele-ophthalmology set-up for making a presumptive diagnosis and planning further management of adnexal and orbital diseases based on live interaction and digital still images of the patients.

Telemedicine is an interesting tool for providing quality medical care among the rural masses especially in remote areas. Telemedicine is defined as incorporation of telecommunication technologies into curative medicine.[1] The efforts to fuse telecommunication technology into medicine have been going on since sometime with the earliest efforts at Nebraska psychiatric institute in 1972.[2]

Ophthalmology as a specialty is readily adaptable to a tele-health delivery system as most of the diagnostic instruments can be easily adapted to mount still and video cameras. Ophthalmologists regularly analyze photographic images to make diagnostic inferences for a number of eye conditions as part of their standard practice.

In India, Tele-ophthalmology is still in its early days with the earliest role in providing comprehensive eye care still under study. We studied the role of tele-ophthalmology in the diagnosis of adnexal and orbital diseases.

## Materials and Methods

A retrospective chart review was done as part of the rural tele-ophthalmology project of a tertiary eye care hospital. This project involved linking remote areas to a specialized base hospital using tele-ophthalmological means. At the time of conducting this study, six remote villages located at an average distance of 198 miles (range 148-229 miles) from the base hospital had been covered in the project. These villages had no ophthalmologists and the nearest ophthalmologist was at an average distance of 30 miles (range 19-41 miles).

**Figure 1:**
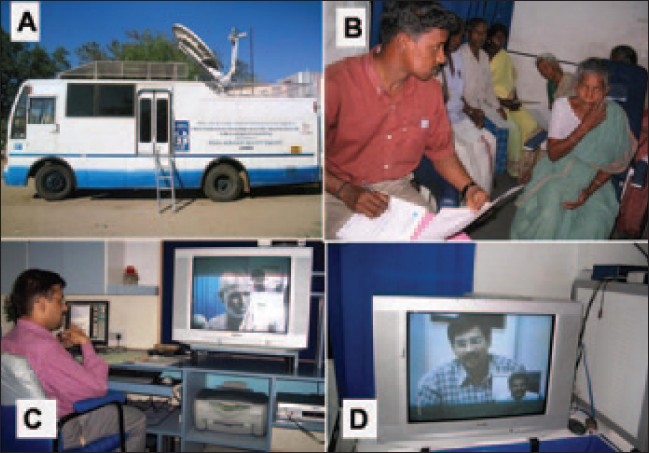
Set up of the rural tele-ophthalmology project. A: Mobile van with dish antenna for satellite connectivity. B: Optometrist screening patients in the rural camps. C: Ophthalmologist at base hospital having real time interaction with the patient. D: The ophthalmologist as seen at the rural end of the teleconferencing link

A customized mobile van with an in-built ophthalmic examination facility having satellite connectivity provided by Indian Space Research Organization (ISRO) along with a social worker and an optometrist visited the selected sites [Fig. 1]. Patients underwent a preliminary screening for ophthalmic diseases at camps organized at these villages. The visual acuity was measured using logMAR chart. Anterior segment was evaluated with a hand-held (Heine HSL 100 CE) slit-lamp and measurement of intraocular pressure was performed with a Schiotz tonometer. External photographs were taken using a digital camera having a resolution of 3.2 megapixel (Canon USA Inc). Photographs were also taken using the slit lamp imaging system, which provided 96-pixel/inch resolution (Appasamy Associates, Chennai). After pupillary dilatation, a single 45° digital fundus photograph centered midway between the center of the macula and the disc was taken with Topcon TRC NW 100 non-mydriatic camera (Topcon, Tokyo, Japan).

**Figure 2:**
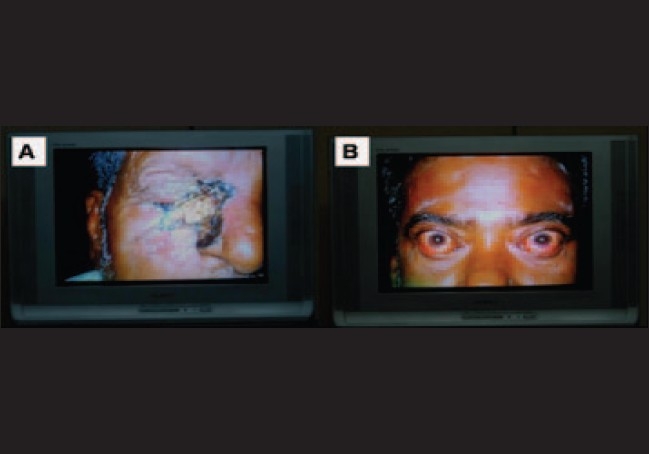
Images as visible to the ophthalmologist at the base hospital through store and forward technique. A: Basal cell carcinoma of the lower lid. B: Thyroid eye disease.

These images were converted to digital imaging and communications in medicine (DICOM) standard and transferred to the base hospital by a satellite link using VSAT (very small aperture terminal) hardware by store and forward technology. The transmission rate was 384 kilobytes per second (kbps). At the base hospital, ophthalmologists studied the photographs and clinical data provided [Fig. 2]. Real time interaction with the examining optometrist as well as the patients was then established using the videoconferencing system (Sony, Tokyo, Japan). The examining optometrist then carried out any further examination felt necessary by the ophthalmologist. Medical treatment advised was then dispensed in the outreach facility and patients needing further investigations or surgical treatment were referred to the base hospital.

## Results

**Figure 3:**
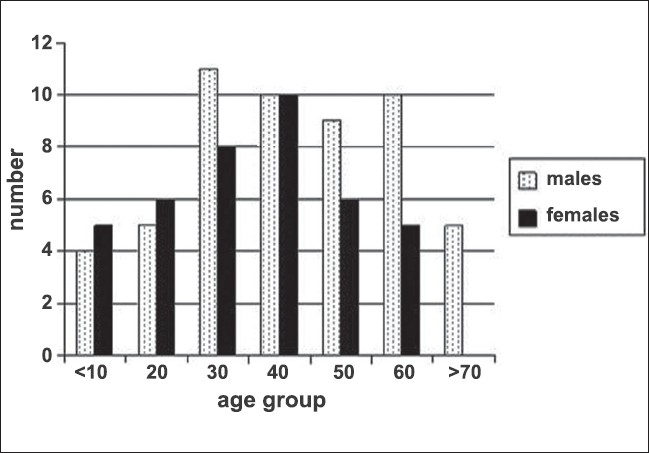
Age wise distribution of patients showing maximum number of patients in economically productive age group

**Figure 4:**
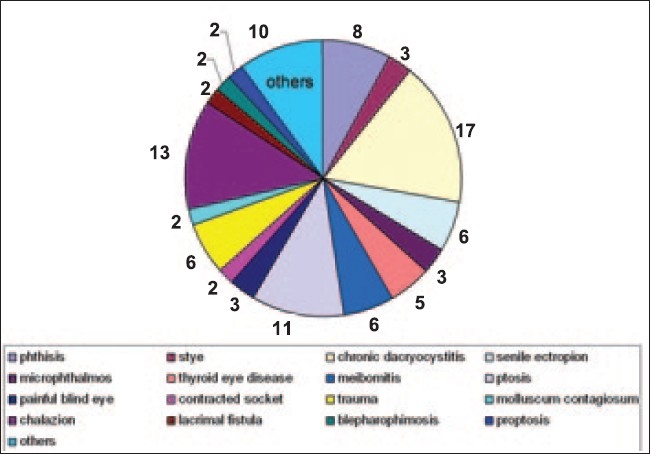
Anatomical distribution of the adnexal and orbital diseases showing maximum involvement of eyelids

Twenty two thousand forty three patients were examined in eye camps as part of the rural tele-ophthalmology project of a tertiary eye care hospital. Out of these, 3497 (15.8%) patients were screened using a combination of store-forward and real time techniques. Out of the 3497 patients, 1998 (57.1%) had refractive errors, 1005 (28.7%) had cataract, 101 (2.88%) had diseases pertaining to eyelids, adnexa and orbit, 70 (2%) had glaucomatous optic discs and 63 (1.8%) had retinal problems. Of those who presented with diseases pertaining to eyelids, adnexa and orbit, most of the patients (38.6%) were in the age between 21 to 40 years [Fig. 3]. There were 58 male and 43 female patients. Sixty-two out of 101 (61.28%) patients had surgically treatable diseases whereas medically manageable diseases were seen in 13 out of 101 (12.8%) patients. The break-up of diagnoses is given in Table 1. Most commonly seen problems were pertaining to lids with 48 of 101(47.52%) patients having infective or non-infective diseases of the eyelids [Fig. 4]. Cosmetic problems like phthisis bulbi requiring simple interventions like fitting of a prosthetic shell were seen in 8 of 101 (7.9%) patients. Eighteen of 101 (17.8%) patients were advised further investigations and evaluation. Patients having trauma (5.9%) and thyroid eye disease (4.9%) commonly required further referral for investigations like thyroid function tests, ultrasound or CT scan. Twenty six out of 101 (25.7%) patients had potentially sight and life threatening problems [Table 2]. They were referred to the base hospital for further management.

**Table 1:** Distribution of the diseases as diagnosed by tele-ophthalmology

Advice	Disease (n)	Total (%)
Cosmetic	Phthisis (8)	8 (7.9)
Medical treatment	Preseptal cellulitis (1), Meibomitis (6), Molluscum contagiosum (2), Stye (3) Acute dacryocystitis (1)	13 (12.8)
Surgery	Blepharoptosis (11), Lid Coloboma (1), Facial nerve palsy (1), Lacrimal Mucocele (1) Chalazion (13), Chronic dacryocystitis (17), Painful blind eye (3), Entropion (1), Lacrimal Fistula (2), Cicatricial ectropion (1), Senile ectropion (6), Basal cell carcinoma (1), Contracted-socket (2), Blepharophimosis (2)	2 (61.28)
Investigations	Microphthalmos (3), Trauma (6), Proptosis (2), Thyroid eye disease (5), Fronto-ethmoidal Osteoma (1), Ocular Myasthenia (1)	18 (17.8)

**Table 2:** Potentially sight and life threatening diseases diagnosed by tele-ophthalmology and their further management (n = 26)

Management advice	Diseases	Total
Medical treatment	Preseptal cellulites (1), Acute dacryocystitis (1)	2
Surgery	Facial nerve palsy (1), Entropion (1), Cicatricial ectropion (1), Senile ectropion (6), Basal cell carcinoma (1)\	10
Investigations	Trauma (6), Thyroid eye disease (5), Fronto-ethmoidal Osteoma (1), Proptosis (2)	14

## Discussion

Telemedicine is an effective fusion of information and communication technology with medicine for meeting the challenge of providing quality eye care among the rural population, especially in remote areas.

Telemedicine had its early roots in the USA with the initial use of a closed circuit TV system to provide a routine distance education and tele-consultation facility between Nebraska psychiatric institute and a remote state mental hospital.[2] In a Norwegian study it was noted that the diagnostic quality of tele-consultation equalled the face-to-face consultations, patients got quality medical care in their own place and the telemedicine saved the transport costs and was very effective.[3]

While most telemedicine models reported earlier used internet to transmit images, we preferred to use satellite mode in India.[4,5] The reason for not using internet was the lack of necessary internet connectivity in the villages. The technical working group (TWG) for standardization on telemedicine in India recommends that satellite link is the best option to connect a remote site with high or flexible bandwidth pipe in the shortest possible time.[6] We have earlier reported the use of telemedicine in diabetic retinopathy screening and have looked into the efficacy, cost effectiveness, patient satisfaction and photographic technique.[7,9]

In our study most of the patients belonged to the economically productive age group of 21 to 40 years. Since these patients are the primary wage earners of the family, they are reluctant to leave their job and come to specialized medical centres for treatment. Though a majority of the patients (61.28%) required surgical intervention, 12.8% of patients could be treated right at their doorsteps by medical means.

Tele-ophthalmological means have mainly been used in screening of retinal diseases or glaucoma.[10–13] Telemedicine has also been used satisfactorily in clinical assessment of the ocular surface.[14] In our study, using tele-ophthalmological means it was possible to satisfactorily obtain images and have live interaction with clarity and resolution high enough to diagnose the patients of adnexal and lid diseases. In patients with orbital diseases also, preliminary diagnoses could be made using tele-ophthalmology.

This study revealed that about 25.7% of patients were living with potentially sight threatening diseases without any access to specialized medical care. The importance of tele-ophthalmology in creating awareness about these diseases and reducing avoidable morbidity is highlighted.

The limitation of our study was that direct confirmation of the diagnoses made using tele-ophthalmology setup was available in those cases only that were referred to the base hospital for further intervention. However since we wanted to study if it is possible to make a diagnosis of adnexal and orbital diseases using tele-ophthalmology (as has been done for other diseases like diabetic retinopathy, glaucoma etc), hence the data studied was limited upto the time of diagnosis and prescription of treatment.[10–13]

This study was conducted in rural and remote areas that do not have access to quality medical care. This study revealed that a large number of patients are living in the remote areas with undiagnosed diseases that can be treated adequately by timely detection and appropriate therapy. It also highlighted the need for health care delivery services in these areas that can be effectively provided by tele-health services, bringing the quality medical care to the doorsteps of the patients from these remote areas.
